# Revealing Lunar Far-Side Polarization Characteristics via FeO Abundance Distribution Correlations with Ground-Based Polarimetric Data

**DOI:** 10.3390/s25185666

**Published:** 2025-09-11

**Authors:** Hanlin Ye, Weinan Wang, Jinsong Ping, Yin Jin

**Affiliations:** 1 International Center for Climate and Environment Sciences, Institute of Atmospheric Physics, Chinese Academy of Sciences, Beijing 100029, China; yehl@radi.ac.cn; 2 National Astronomical Observatories, Chinese Academy of Sciences, Beijing 100012, China; wangwn@mail.iggcas.ac.cn; 3 Institute of Geology and Geophysics, Chinese Academy of Sciences, Beijing 100029, China; 4 University of Chinese Academy of Sciences, Beijing 101408, China; jinyin22@mails.ucas.ac.cn; 5 Key Laboratory of Digital Earth Science, Aerospace Information Research Institute, Chinese Academy of Sciences, Beijing 100094, China

**Keywords:** lunar far side, polarization characteristics, degree of polarization, FeO abundance

## Abstract

Due to the tidal locking, the far side of the Moon is permanently turned away from the Earth. Its polarization characteristics are still poorly understood, limiting our knowledge of material composition and evolution. Previous studies have indicated a correlation between the distributions of degree of polarization (DOP) and the iron oxide (FeO) abundance on the Moon, suggesting a new approach to infer the polarization characteristics of the lunar far side from FeO abundance distribution. Three critical issues have been analyzed: (1) A linear regression model between DOP and FeO abundance is proposed based on control points from ground-based near side polarization images. (2) The DOP distribution of the lunar far side is estimated, based on the established model, revealing significant hemispheric differences in polarization characteristics. (3) The relationship between DOP and lunar phase angle is examined, with the fitted values demonstrating strong agreement with the observations in both magnitude and variation trend. These insights offer valuable guidance for comprehensive polarimetric studies of the Moon.

## 1. Introduction

Lunar exploration, serving as the starting point of human exploration in the solar system, has significantly promoted understanding of the Moon, the Earth, and other celestial bodies in the solar system [1,2,3,4,5,6,7].

The purpose of lunar exploration is to understand the origin and evolution of the Moon, as well as to describe the state of the lunar surface and its interactions with the space environment [8,9]. Currently, the mainstream methods of lunar exploration are mainly divided into two categories: in situ exploration and remote sensing [9]. In situ exploration relies on landers and lunar rovers to conduct on-site scientific investigations on the lunar surface [10]. Through techniques such as drilling, sampling, and spectral analysis, it accurately obtains key data on the composition of lunar rocks and minerals, as well as geological structures, providing essential evidence for studying the material composition and evolutionary history of the Moon [11,12,13]. Remote sensing, utilizing non-contact observation, can be further categorized into two types. The first type involves circumlunar orbiters equipped with advanced payloads, such as high-resolution cameras and multispectral imagers [14,15], which conduct comprehensive observations of the Moon, systematically mapping topographical features and tracking the distribution patterns of minerals. The second type employs ground-based telescopes to monitor the Moon continuously from Earth [16] using visible light, infrared, and radio wave bands [17,18,19,20]. Despite the interference from the Earth’s atmosphere, these telescopes have unique advantages in capturing long-term changes and the dynamic evolution of the whole lunar disk. However, constrained by the tidal locking effect, ground-based telescopes are not only unable to observe the entire far side of the Moon but also struggle to obtain a complete view of the lunar polar regions. The inherent blind spots in ground-based observations severely restrict systematic research on the far side of the Moon.

In the field of remote sensing technologies, polarization detection captures the polarization state information of the reflected light from the lunar surface [21,22,23]. This enables it to obtain microscopic features such as the size of material particles, surface roughness, and crystal structures, which are difficult to reveal through traditional spectral and imaging techniques [24,25]. These data can be used to refine three-dimensional optical models of lunar surface materials [26], allowing for precise inversion of the thickness of lunar regolith and the distribution of mineral components [23,27,28]. As a result, polarization detection significantly promotes in-depth understanding of the material composition of the lunar surface, geological evolution mechanisms, and the interactions with the space environment, providing a new technical perspective and scientific basis for deciphering the formation and evolutionary history of the Moon.

Currently, the scientific community has conducted relatively systematic research on the polarization characteristics of the near side of the Moon, accumulating abundant achievements in aspects such as the polarization mechanism of reflected light and the polarization patterns of mineral components. Previous studies have mainly carried out systematic studies on the correlation between the DOP and albedo of different lunar regions under different lunar phase angle conditions through ground-based polarization observations. Kohan [29] investigated the polarization characteristics of the lunar surface by measuring the DOP and the angle of orientation of the plane of polarization of reflected light under varying phase angles. Dollfus [27] utilized imaging polarimetry to study the polarization characteristics of several individual regions on the lunar surface, constructed polarization degree functions, and explored their relationships with phase angle, albedo, surface roughness, and particle size. Jeong et al. [30] conducted multiband polarimetric observations across the entire lunar nearside, analyzing the spatial distribution and interrelationship between surface albedo and the maximum DOP in various regions. Steele et al. [31] conducted polarization measurements during the first 15 minutes of totality of the 16 May 2022 lunar eclipse and attributed the observed polarization primarily to scattering in the Earth’s atmosphere rather than to the lunar regolith. Arnaut et al. [32] analyzed the small-scale structure of the lunar regolith by combining telescopic multispectral UBGRI polarimetric data with Chandrayaan-1 M3 reflectance measurements to investigate correlations with lunar terrain and to stimulate further research on fine structures of the lunar surface. Venkatesulu et al. [33] conducted high-resolution measurements of the Moon’s polarization, providing disk-resolved polarization images across a wide range of lunar phases, offering new insights into the spatial distribution and phase angle dependence of moonlight polarization. Kreslavsky et al. [34] presented preliminary analyses of PolCam data from the Danuri mission, showing that some small, young lunar craters exhibit high polarization due to coarse ejecta that have not yet been reworked into mature regolith. Venkatesulu and Shaw [21] used a division-of-focal-plane polarization camera to record polarization images of the Moon at 40 phase angles, and calculated disk-averaged DOP. They found higher polarization in lower reflectivity regions, higher polarization in waning phases than waxing phases, with the results consistent with previous studies. These studies have not only verified the logarithmic linear relationship between the DOP and albedo in Umov’s law [35,36], but also deeply analyzed the spatial distribution law of polarization parameters and albedo, and their differential correlations with albedo in mare and plateau regions, providing a solid foundation for establishing a quantitative relationship between the DOP or other polarization parameters and albedo on the lunar surface.

However, there are still significant gaps in the systematic observation and study of the polarization characteristics of the lunar far side. The core bottleneck lies in the insufficient data acquisition. Affected by the Earth–Moon tidal locking, the far side of the Moon always faces away from the Earth, making it difficult for ground-based telescopes to break through the limitations of the observation blind zone. In terms of orbiters, only the Korean Pathfinder Lunar Orbiter (KPLO) has conducted polarization detection of the lunar far side worldwide [37,38]. Constrained by the satellite’s observation geometry, this orbiter can only perform measurements during specific orbital phases. As a result, it struggles to obtain spatial continuity data under different lunar phases in the same way that ground-based telescopes can continuously observe the near side of the Moon.

The lack of such data prevents a systematic analysis of the polarization characteristics on the far side of the Moon, creating significant blind spots in our understanding of the geological evolution process, much like missing the ‘other half of the puzzle’. Filling these knowledge gaps will not only refine the theoretical framework of the Moon’s origin and evolution but also provide essential scientific support and decision-making basis for future deep-space exploration projects, including the scientific goal design of far-side lunar sample return missions and the geological safety assessment of potential lunar base sites.

As an important dimensional property of light, polarization is closely related to the crystal structure or subsurface structure of minerals. In the field of Earth observations, polarization observation technology has been proven to effectively distinguish the states of different minerals on Earth [28]. For the Moon, lunar regolith particles contain more information about basic components. FeO is widely regarded as a representative chemical component of lunar regolith, whose abundance reflects both geological evolution and space weathering processes, while also exerting a strong control on the optical scattering properties that determine polarization. Wang et al. [39] attempted to explore the relationship between the DOP and the FeO abundance in lunar regolith.

According to Wang et al. [39], this study constructs a relationship model between the DOP and FeO abundance by integrating ground-based polarization observation data and validates its effectiveness. On this basis, the model is used to retrieve the FeO abundance in the far-side lunar regions, revealing the spatial distribution differences and regularities. This research not only provides a new approach to uncover the polarization characteristics of the lunar far side, filling the research gap in this field, but also establishes a methodological framework for exploring the application potential of ground-based polarization observations in lunar macro-scale remote sensing.

Distinguished from prior research, this study analyzes the relationship between the DOP and FeO abundance by extracting feature points (such as lunar craters and mountain ridges) from polarization images. Three key contributions are made to the understanding of lunar polarization characteristics. First, control points selected from the lunar near side are used to establish a linear relationship between the DOP and FeO abundance. This relationship accurately captures the spatial variation in the observed DOP and shows consistency in high-DOP regions. Second, leveraging this relationship, we derive the DOP distribution of the lunar far side for the first time using FeO abundance data, revealing large-scale hemispheric differences in polarization that reflect the compositional differences between the near side and far side of the Moon. Third, we examine the dependence of DOP on lunar phase angle. We demonstrate that the simulated and observed DOP values of sunlit regions agree strongly in both magnitude and phase-dependent variation, which highlights the reliability of our approach and lays a foundation for extending ground-based polarimetric methods to broader lunar remote sensing applications.

This article is organized into several main parts. The first part is the Introduction. Section 2 provides a detailed description of the polarization observation data and FeO abundance data used in this study. Section 3 presents the preprocessing workflow, which mainly includes offset subtraction, dark-field correction, and flat-field correction, and also describes the extraction of control points and image registration. Section 4 then analyzes the spatial distribution of the lunar far-side DOP derived from the linear relationship between DOP and FeO abundance, and the variation in DOP with lunar phase angle. Section 5, in the context of the development of lunar remote sensing, discusses the implications of the current lack of lunar far-side polarization information and provides advice for future lunar polarization studies. Finally, Section 6 summarizes the main conclusions of this study and highlights its scientific significance.

## 2. Data

### 2.1. Polarization Data from DoFP Camera

**Figure 1:**
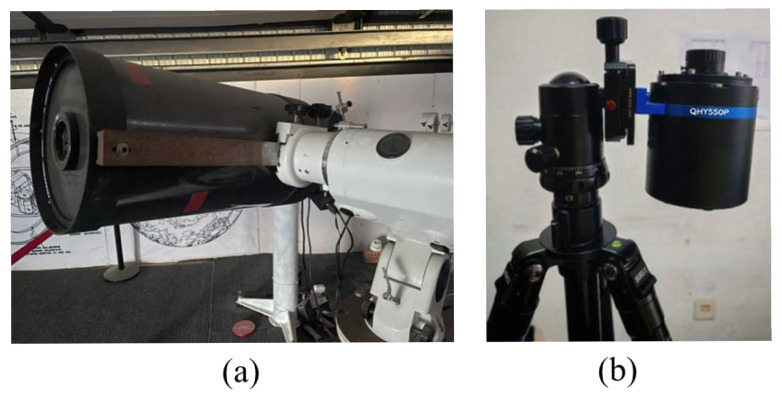
(**a**) Schmidt Cassegrain Telescope. (**b**) The QHY550P DoFP camera to be mounted on the telescope.

From August 2022 to April 2023, multiple white-light polarization observations of the entire lunar nearside were conducted at the Gaoyazi Observatory in Xinjiang [22], with a motorized EQ5 equatorial(manufactured by Pacific Telescope Corp., Richmond, BC, Canada) mount employed to track lunar motion. Situated at (43.5219° N, 87.6005° E) in Dabancheng District, Urumqi, Xinjiang, the Gaoyazi Observatory benefits from an exceptional night-sky background and optimal astronomical observation conditions. The observations utilized a Celestron C925 Schmidt–Cassegrain telescope (manufactured by Celestron, Torrance, CA, USA) with a 235 mm aperture, 2350 mm focal length, and F/10 focal ratio, as shown in Figure 1. This telescope features a cost-effective design without polarization elimination. A QHY550P focal-plane polarization (DoFP) imaging camera (manufactured by QHYCCD, Shenzhen, China) with 12 effective bits of analog-to-digital (A/D) sampling depth was used. The camera has a full resolution of 2460 × 2070, covers the wavelength range of 400–800 nm, and offers a field of view (FOV) of 12.43′ × 10.37′. A multi-directional on-chip polarizer is integrated directly above the photodiodes of the sensor chip.

The DoFP camera represents an advanced technology that integrates image sensing with polarization detection. By embedding micro-polarizers onto a complementary metal-oxide-semiconductor (CMOS) image sensor (manufactured by Sony, Tokyo, Japan), this technique enables simultaneous acquisition of polarization information and intensity images. During semiconductor fabrication, linear grating micro-polarizers oriented at 0°, 45°, 90°, and 135° are directly etched onto the photosensitive chip, endowing each pixel with polarization-sensing capability. The pixel-level micro-polarization array, covering the entire sensor area, is co-packaged with a micro-mirror array to form a complete DoFP camera system.

As a pioneering technology, the DoFP camera achieves pixel-level integration of four-directional polarizers (0°, 45°, 90°, 135°), significantly enhancing observation accuracy and efficiency. The sensor is constructed by depositing a micro-polarization array onto the pixel surface, over which a micro-mirror array is packaged. This four-directional focal-plane polarization sensor allows the pixel array to output multi-polarization images. Since the polarization array covers the entire pixel grid, extracting pixels of a specific orientation during imaging yields direction-specific polarization images. This capability improves imaging contrast, depth perception, and physical information extraction efficiency. With a simple system architecture and high stability, the DoFP camera meets real-time detection demands for dynamic targets.

During the observation, the camera’s gain was set within the 1–50 range and remained constant throughout the night. To determine optimal exposure time and gain parameters, the average count value of images was maintained between 40,000 and 55,000. Given the lunar phase variations, distinct exposure times were applied: each image frame typically had an exposure time of 20–30 ms. Prior to polarization observations, bias, dark-field, and flat-field images were selected as auxiliary images for instrumental calibration. Additionally, specific polarization standard stars were used to measure and correct the camera’s instrumental polarization.

In this study, data from another sensor were also utilized [21]. This sensor is primarily a commercial DoFP camera (FLIR Blackfly BFSU3-51S5P-C, Wilsonville, OR, USA), which can simultaneously capture panchromatic images in the 400–1000 nm broadband at four polarization states of 0°, 45°, 90°, and 135°. The detector is equipped with an array of wire-grid polarizers arranged in 2 × 2 super-pixels, where the four sub-pixels of each super-pixel are oriented to measure intensities in the four polarization states.

The camera was deployed at the focus of two imaging systems. One is a 300 mm telephoto lens, including a Nikon lens(manufactured by Nikon Corporation, Otawara, Japan) with a maximum aperture of f/4 (AF–S 300 mm, f/4) and a Nikkor lens (manufactured by Nikon Corporation, Tochigi Prefecture, Japan) with a maximum aperture of f/4.5 (Nikkor*ED 300 mm 1:4.5 IF AIS), both set to f/11. When equipped with a 300 mm lens, each sub-pixel corresponds to an instantaneous FOV (IFOV) of 0.000669° (11.7 μrad). The other is a Celestron Schmidt–Cassegrain telescope with a f-number of f/10 and a focal length of 2.032 m, and the IFOV of each sub-pixel is 0.000102° (1.78 μrad).

For calibration, an integrating sphere (Labsphere USLR-V12F-NDNN-P, manufactured by Labsphere, North Sutton, NH, USA) was used as the illumination source, and a high-extinction-ratio wire-grid polarizer (Meadowlark Optics Versalight, manufactured by Meadowlark Optics, Frederick, MD, USA) on a precision rotation stage (Newport RV160CC, manufactured by Newport Corporation, Irvine, CA, USA) was employed to control the polarization state of the incident light. Using illumination provided by the integrating sphere, rotate the polarizer to four angles (0°, 45°, 90°, and 135°), record images under these four calibration scenarios, respectively, and establish a conversion matrix between the incident linear polarization Stokes vector and the measured Stokes vector to achieve polarimetric calibration.

### 2.2. Lunar Surface FeO Abundance

**Figure 2:**
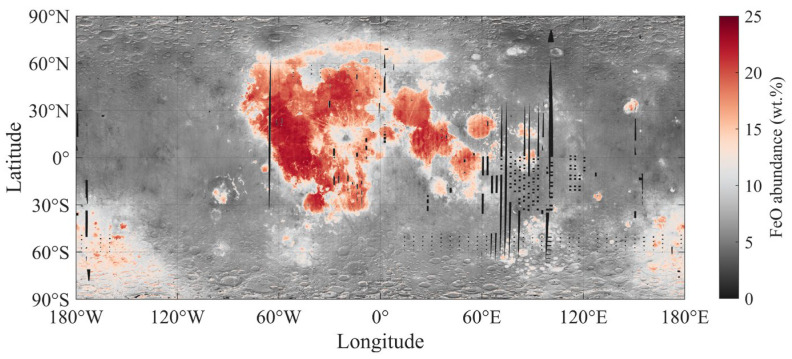
Global distribution map of lunar regolith FeO abundance.

In this study, lunar regolith FeO abundance is used to establish the correlation between FeO abundance and DOP at specific lunar positions, and to derive far-side polarization values via this correlation. This study employs the lunar regolith FeO abundance proposed by Meng et al. [40,41], which is derived from Chang’E-2 Microwave Sounder (CELMS) data and a back propagation neural network (BPNN) method, with a final spatial resolution of 0.1° × 0.1°, as shown in Figure 2.

The core advantages of this proposed method lie in high-precision inversion of lunar regolith FeO abundance through multi-source data fusion and neural network modeling: integrating four-channel microwave brightness temperature (TB) data at 3.0–37.0 GHz from CELMS (covering meter-scale penetration depth) with LOLA surface slope data, terrain-induced microwave radiation interference is corrected by slope adjustment, which reflects regolith internal composition more effectively than single optical data (only representing micron-scale surface information). A three-layer BPNN optimizes node weights via the generalized delta rule, effectively capturing the non-linear segmentation of TB-FeO abundance around the 16 wt.%, with 1000 iterations at a 5% error threshold, ensuring prediction accuracy. The inversion results highly coincide with Clementine UV-VIS and Lunar Prospector (LP) Gamma Ray Spectrometer (GRS), which was developed by Los Alamos National Laboratory, data in the spatial distributions of Maria and highlands [42,43], and also coincide well with LP GRS in terms of values. Meanwhile, the 0.1° × 0.1° global map meets the spatial resolution requirements of ground-based observations.

## 3. Method

### 3.1. Data Preprocessing

After acquiring image data using the DoFP camera, data preprocessing is a critical step to ensure image quality and the accuracy of subsequent analysis. For our specific QHY550P DoFP camera, the raw data require preprocessing procedures, primarily including bias subtraction, dark-frame correction, and flat-field correction. To address the limitations of the DoFP camera, we applied the image interpolation method to mitigate the effects of reduced spatial resolution and instantaneous IFOV errors. Due to the limited FOV of the camera, which made it impossible to capture the entire lunar disk in a single frame, we built a rigorous geometric imaging model based on the camera’s imaging characteristics to help align and combine multiple sub-images into a corrected full-Moon image. The following sections describe each of these processes in detail.

(1) Bias, dark frame, and flat-field Corrections

To reduce thermal noise in the DoFP camera and minimize systematic errors from the imaging system, all Stokes parameters were processed individually. This procedure included corrections using bias subtraction, dark-frame, and flat-field corrections. The bias image was used to compensate for fixed-pattern noise and electronic bias present during image acquisition. The dark-frame image corrected thermal noise resulting from internal thermal motion within the camera, and was recorded with the same exposure time as the observations. The flat-field image was applied to correct for non-uniform illumination caused by optics such as mirrors, filters, or other optical components, and for pixel response non-uniformity inherent to the CMOS sensor. One hundred out-of-focus images of an integrating sphere were averaged, and their pixel-wise multiplicative inverse was computed. The resulting array was then normalized to a mean of one to generate the flat-field correction matrix. Flat-field correction was subsequently applied to each raw image through pixel-wise multiplication with this matrix, yielding corrected images in DN units.

In addition, each image was divided by its respective exposure time. This conversion transforms the raw images into count rate images, correcting for intensity variations among images arising from differences in exposure time.

(2) Image interpolation and processing

DoFP cameras have several inherent limitations. These include IFOV errors caused by pixel-level misalignment among the different polarization filter elements within each super-pixel. In addition, the pixel response exhibits non-uniformity in amplitude, uncertainty in angle reconstruction, and misalignment between pixels. As a result, after preprocessing the four raw polarization intensity images (corresponding to 0°, 45°, 90°, and 135°), we applied bicubic spline interpolation to mitigate the effects of spatial resolution degradation and IFOV errors [44,45].

Bicubic spline interpolation is a linear interpolation method that uses the grayscale values of neighboring pixels to generate smooth transitions, producing continuous curves and surfaces. This results in smoother images with preserved details and reduced aliasing.

To begin, pixels corresponding to the same polarization direction were extracted from the raw image, forming four sub-images, with each one representing a single polarization angle and having one-quarter the size of the original image. Then, bicubic spline interpolation was applied individually to the four sub-images to generate interpolated images: $I_{0^{\circ }}$, $I_{45^{\circ }}$, $I_{90^{\circ }}$, and $I_{135^{\circ }}$, which match the original image size in dimensions.

(3) Polarimetric Calibration

By multiplying the Stokes vector calculated from the raw image data with a pre-determined polarimetric system matrix, we obtained the polarimetrically corrected Stokes vector.

(4) Background subtraction

The corresponding background intensity values in each of the four polarization sub-images were subtracted to effectively mitigate the impact of skylight and instrumental scattering on the polarization parameters. Sky background regions near the edge of the lunar disk were manually selected.

(5) Edge artifacts subtraction

To reduce the impact of edge artifacts on subsequent parameter calculations, we applied an edge-smoothing technique to each polarization sub-image using the bilinear interpolation method introduced by Ratliff et al. [45], which is commonly used in polarization image processing.

(6) Extraction of Stokes parameters and mask generation

First, based on the following Equation, the Stokes parameters and DOP were calculated using the four processed polarization sub-images. First, the Stokes parameters and DOP were calculated using the four processed polarization sub-images based on the following equation. A thresholding operation was then applied to the intensity I image to generate a binary mask, from which the largest connected component was extracted to define the lunar pixel mask. This mask was used to isolate pixels corresponding to the lunar region, enabling the computation of spatially averaged DOP values over the Moon’s disk.

(1) $$I=\frac{I_{0^{\circ }}+I_{45^{\circ }}+I_{90^{\circ }}+I_{135^{\circ }}}{2}$$

(2) $$Q=I_{0^{\circ }}-I_{90^{\circ }};\;U=I_{45^{\circ }}-I_{135^{\circ }}$$

(3) $$DoP=\frac{\sqrt{Q^{2}+U^{2}}}{I}$$

Here, $I_{0^{\circ }}$, $I_{45^{\circ }}$, $I_{90^{\circ }}$, and $I_{135^{\circ }}$ represent the sub-images corresponding to polarization directions of 0°, 45°, 90°, and 135°, respectively. The Stokes parameters I, Q, and U can then be computed using Equations (2) and (3).

(7) Geometric correction and mapping between image points and object points

Due to the limited FOV and spatial resolution of the DoFP camera, images of the Moon must be mosaicked from multiple frames. Therefore, the images were geometrically corrected and projected onto the Moon-Centered Moon-Fixed (MCMF) coordinate system to facilitate further analysis. A rigorous geometric imaging model was employed to establish the mathematical relationship between object points and image points, as described by the following equation:

(4) $${\left[\begin{array}{c} X\\ Y\\ Z \end{array}\right]=\left[\begin{array}{c} X_{S}\\ Y_{S}\\ Z_{S} \end{array}\right]+{m(R_{SCRS}^{MCMF}R_{ITRS}^{SCRS}R_{J2000}^{ITRS}R}_{Body}^{J2000})R}_{Camera}^{Body}\left[\begin{array}{c} x\\ y\\ f \end{array}\right]$$

where ${\left[X\;Y\;Z\right]}^{T}$ and ${\left[X_{S}\;Y_{S}\;Z_{S}\right]}^{T}$ represent the coordinates of the ground point and the projection center, respectively, in the Moon-Centered Moon-Fixed (MCMF) coordinate system. The vector ${\left[x\;y\;f\right]}^{T}$ denotes the image point in the camera coordinate system. $R_{Camera}^{Body}$ is the rotation matrix from the camera coordinate system to the body coordinate system. $R_{Body}^{J2000}$ converts from the body coordinate system to the J2000 coordinate system. $R_{J2000}^{ITRS}$ is the rotation matrix from the J2000 coordinate system to the International Terrestrial Reference System (ITRS). $R_{ITRS}^{SCRS}$ converts from ITRS to the selenocentric reference system (SCRS), and $R_{SCRS}^{MCMF}$ transforms from SCRS to MCMF. The scalar m is a scale factor.

This model corrects pointing errors using the exterior orientation matrix, and mitigates image distortion caused by principal point shifts and focal length effects through a third-order polynomial fit. With the accurate image-to-ground mapping in place, image points were projected onto the SCRS, facilitating the mosaicking of lunar polarization data.

**Figure 3:**
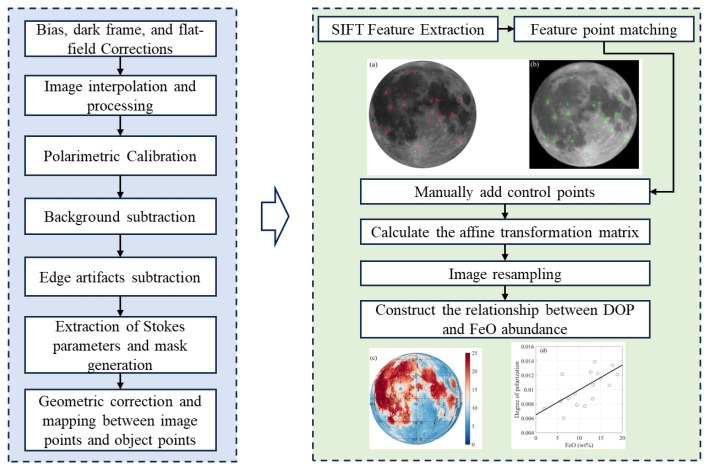
The flowchart for raw image preprocessing. (**a**) Lunar albedo image, with red markers indicating the locations of control points. (**b**) Lunar polarization image, with green markers indicating the locations of control points. (**c**) Distribution of FeO abundance on the lunar near side. (**d**) Linear relationship between DOP and FeO abundance derived from the control points.

The preprocessing procedure for each raw image is illustrated in Figure 3.

### 3.2. Image Registration

Based on the processed full-disk lunar polarization images, image registration with the orthorectified lunar albedo image is first conducted to accurately assign lunar longitude and latitude coordinates to each polarization pixel, thereby enabling a one-to-one correspondence with the FeO abundance on the lunar surface.

As shown in Figure 3, during the image registration process, a set of corresponding control points is first selected between the orthorectified lunar albedo image and the lunar polarization image, establishing the foundation for subsequent spatial transformations. To improve matching accuracy and automation, the Scale-Invariant Feature Transform (SIFT) operator is employed to automatically extract feature points from both images [46]. SIFT operates by constructing a scale-space to identify keypoints across images with varying levels of blurring. Specifically, it applies Gaussian filtering to the original image at multiple scales, generating a series of blurred images. The differences between adjacent scale levels are then calculated to construct a difference of Gaussians (DoG) pyramid. Within this pyramid, local extrema are detected across both spatial and scale dimensions to determine the location and scale of keypoints.

To further enhance registration accuracy, additional control points were manually selected to supplement the automatically extracted features. These control points are typically concentrated in regions with distinct textures or structural boundaries, such as the edges of lunar impact craters, mountain ridges, and other geological features, providing high stability and robustness for image matching. In each lunar polarization image, several dozen control points are selected. Feature point matching was performed between the two images based on these combined sets of points. Similarity between feature vectors was evaluated using the Euclidean distance metric. For each feature point in the full-disk lunar polarization image, the corresponding candidate match was identified by locating the feature vector with the shortest Euclidean distance in the orthorectified lunar albedo image. A valid match was confirmed if the distance between two feature vectors fell below a predefined distance threshold.

Once pairs of matching points were obtained, the affine transformation matrix was estimated using the least squares method. This transformation matrix was then applied to map all pixels in the polarization image to the coordinate system of the albedo image, thereby assigning each polarization pixel a unique lunar longitude and latitude coordinate.

Finally, a pixel-level scatterplot was constructed to examine the relationship between the DOP and FeO abundance over the lunar near side. A significant correlation between DOP and FeO abundance on the lunar nearside suggests that the polarization distribution on the far side can be indirectly inferred from FeO abundance, offering a basis for characterizing far-side polarization in the absence of direct observations.

## 4. Results

### 4.1. Analysis of the DOP Distribution

**Figure 4:**
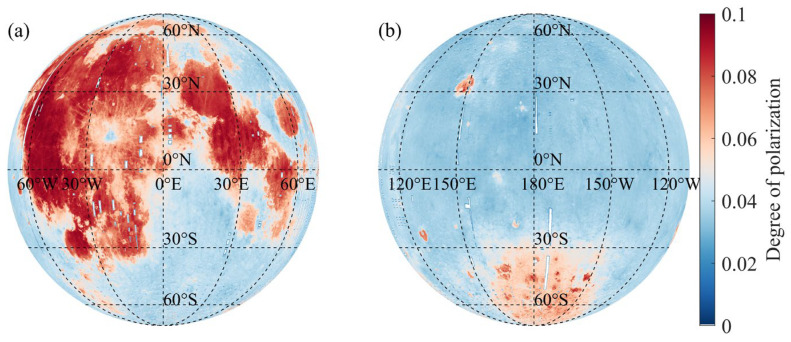
DOP distribution map of the lunar (**a**) near side and (**b**) far side.

Based on the afore-mentioned method, a linear regression model was established between FeO abundance and the DOP. Using this model, a global DOP distribution map of the lunar surface was derived from the polarization image acquired at full moon (Figure 4). Polarization images acquired at the full Moon phase ensure uniform illumination and maximal surface visibility, enabling the extraction of more control points and improving the accuracy of the derived far-side DOP. Notably, the dark blue bands in Figure 4 correspond to regions with missing FeO abundance, where the DOP values could not be retrieved.

As shown in Figure 4a, the DOP distribution on the lunar nearside exhibits distinct regional characteristics. High DOP values are concentrated near the equator and within the longitude range of 30° W to 60° W, especially in lunar mare regions such as Oceanus Procellarum, Mare Serenitatis, Mare Imbrium, and Mare Tranquillitatis. When compared with the lunar albedo image, these areas correspond to low-albedo, darker regions of the surface, yet exhibit higher polarization, which is consistent with the physical mechanism of the Umov effect [47], which states that lower reflectance is typically associated with higher DOP due to increased surface scattering. Several previous missions have conducted in situ investigations of the lunar surface [11,39,48]. We compared our results with these in situ sampling data, as presented in Table 1, and the comparison confirms the consistency between the observed DOP and the fitted DOP.

In Figure 4b, despite the overall lower DOP values on the far side, several localized high-DOP areas are still evident. These include parts of the South Pole–Aitken Basin, Mare Ingenii, and Mare Moscoviense. Compared with the nearside, the DOP values on the far side are more uniformly distributed, showing a spatial pattern broadly consistent with the far-side FeO abundance distribution.

**Figure 5:**
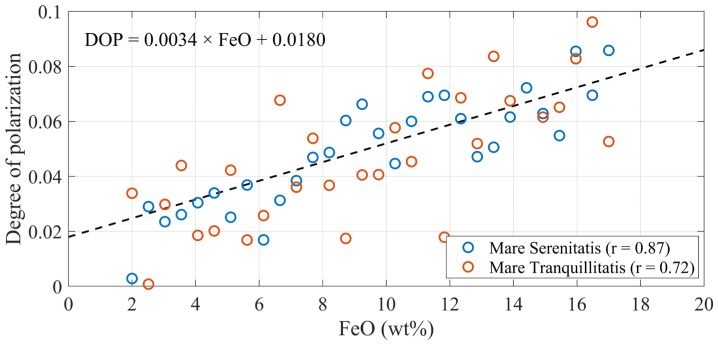
Linear correlation between DOP and FeO abundance, with corresponding control point distributions shown for Mare Serenitatis (Pearson correlation coefficient r = 0.87) and Mare Tranquillitatis (r = 0.72).

Figure 5 presents the linear relationship between DOP and FeO abundance, along with the distribution of control points extracted from the Mare Serenitatis and Mare Tranquillitatis. Overall, DOP exhibits a clear upward linear trend with increasing FeO abundance, indicating that FeO abundance, as a key mineral component of the lunar regolith, has a significant influence on the polarization characteristics of reflected light from the lunar surface. The Pearson correlation coefficient reaches 0.8303 in the Mare Serenitatis and 0.6118 in the Mare Tranquillitatis. The trend appears more stable in Mare Serenitatis, while the data points in Mare Tranquillitatis are more scattered, possibly due to factors such as local topographic variation, errors in FeO abundance, or changing observation conditions, leading to a greater number of outliers from the linear trend.

**Figure 6:**
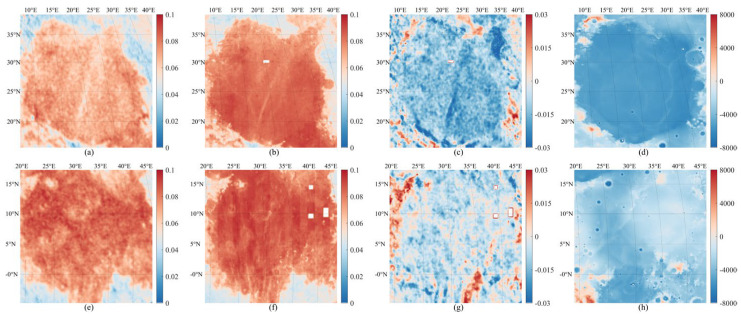
Distribution analysis of DOP and DEM in Mare Serenitatis (**a**–**d**) and Mare Tranquillitatis (**e**–**h**) on the Moon. (**a**,**e**): raw observed DOP images; (**b**,**f**): DOP images fitted based on the linear relationship between FeO abundance and DOP; (**c**,**g**): residual distributions between raw and fitted DOP values (raw minus fitted); (**d**,**h**): DEM maps of the corresponding regions (m).

As shown in Figure 6a,e, the DOP values in Mare Serenitatis and Mare Tranquillitatis are generally high, with particularly pronounced values in the inner parts of these maria, consistent with the elevated FeO abundance in these regions. These high polarization characteristics are closely associated with the basaltic composition of the lunar regolith and exhibit strong regional continuity. Similarly, the fitted DOP maps in Figure 6b,f aexhibit a comparable spatial pattern and effectively capture the overall trend of the original DOP distribution, with a high degree of consistency, especially in regions where DOP values are elevated.

Figure 6c,g further illustrate the residuals between the observed and fitted values. In the Mare Serenitatis, the residuals show a distinct alternation of positive and negative values along the mare boundary, with differences exceeding 0.015 in certain locations. This indicates that the fitting model exhibits deviations in transitional zones, likely due to terrain undulation and discontinuities in FeO abundance distribution. In the Mare Tranquillitatis, the residual distribution is more complex, with clusters of positive and negative residuals in several localized areas, which may result from the combined effects of complex topography and variations in lithological composition.

The digital elevation model (DEM) imagery further confirms the influence of topography on the spatial distribution of DOP. Regions exhibiting high DOP values are primarily located in relatively flat terrains or basin-like depressions, whereas areas with abrupt terrain changes or meteorite crater rims show significant DOP disturbances. Based on the residual maps, it can be inferred that in regions of sharp topographic variation, the observed DOP is influenced by both scattering anisotropy and surface roughness, making it difficult for the linear fitting model to achieve accurate predictions, thus leading to large fitting errors.

### 4.2. Relationship Between the Mean DOP and Lunar Phase Angles

**Figure 7:**
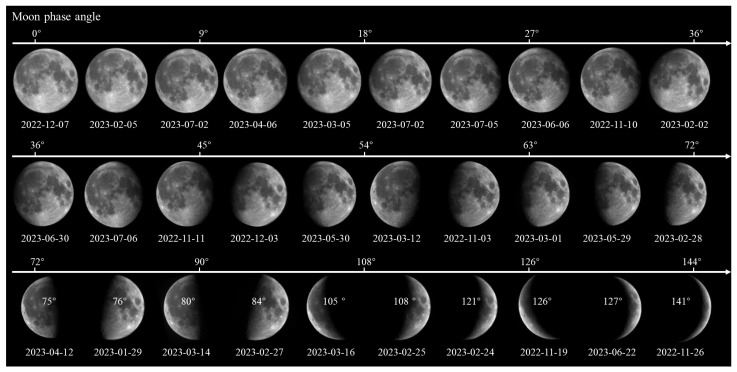
Lunar polarization images under different phase angles acquired between November 2022 and March 2023.

Figure 7 presents a time series of lunar polarization images acquired under varying phase angles, aiming to illustrate the periodic evolution of the Moon’s polarization characteristics. This study selects representative observations from a total of 30 polarization images acquired between November 2022 and March 2023, covering the full lunar phase cycle. Phase angle, representing the relative geometric relationship among the Moon, Earth, and Sun, directly influences the illuminated area of the lunar surface and its reflective properties.

**Figure 8:**
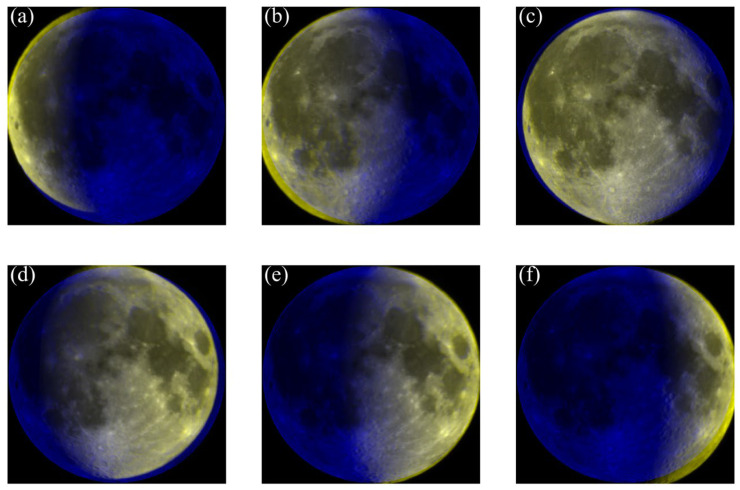
Overlap and registration of lunar polarization images acquired under different phase angles. The blue regions represent the albedo image of the lunar near side, while the yellow areas indicate the polarization images overlaid after registration. Subfigure (**a**–**f**) correspond to phase angles of 108°, 58°, 32°, 49°, 70°, and 105°, respectively.

Figure 8 illustrates the overlap and registration results of lunar polarization images acquired under different phase angles, which are used to analyze the spatial distribution of polarization characteristics throughout the lunar phase cycle. Most images show satisfactory registration results; however, misalignments persist along the lunar limb areas. These inconsistencies are primarily caused by the effect of lunar libration, which results from the Moon’s orbital eccentricity and axial tilt. Libration leads to variations in the visible portion of the near side from Earth at different times, causing slight shifts in the observed areas. Additionally, errors in control point extraction and geometric distortions occurring during image registration may further contribute to local misalignments. The combined influence of libration and registration inaccuracies accounts for the differences observed in the multi-temporal image overlays.

**Figure 9:**
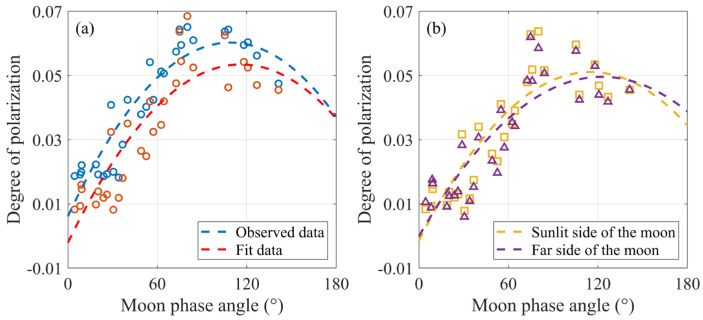
Relationship between lunar DOP and phase angle: (**a**) Mean DOP variation with phase angle in the sunlit lunar near side. Blue scatter points represent measured DOP values; red scatter points represent DOP values derived from the linear relationship with FeO abundance. (**b**) Mean DOP values at different phase angles in sunlit regions of the entire Moon and the lunar far side. Yellow and purple scatter points represent DOP values derived from the linear relationship with FeO abundance.

Figure 9 further illustrates the variation characteristics of the DOP for the sunlit near side, sunlit far side, and the entire sunlit region of the Moon under different phase angles. Figure 9a presents the trend of average DOP variation for the sunlit near side across different phase angles. Overall, the trend reveals that the DOP increases with phase angle, reaching a peak around 100°, followed by a slight decline. This result validates the reliability of using FeO abundance to estimate DOP from two perspectives. First, the observed data and fitting results are in good numerical agreement, particularly within the phase angle range of 30° to 120°, where a high degree of overlap is observed. Second, the fitted curves exhibit similar trends, indicating strong physical consistency and regional applicability of this method on the lunar near side.

As shown in Figure 9b, the DOP values for both regions are generally similar across most phase angles; however, the DOP of the entire sunlit surface is slightly higher than that of the sunlit far side, primarily due to the contribution from the near side. The DOP of the entire sunlit surface reaches its peak at a phase angle of approximately 115°, whereas the peak for the sunlit far side occurs around 123°. The near side of the Moon contains extensive basaltic mare regions characterized by low albedo and high FeO abundance, which enhances the polarization of solar radiation and results in higher DOP values under similar illumination conditions [49]. In contrast, the far side is dominated by highland terrain with lower FeO abundance and rougher surface textures, leading to a comparatively weaker polarization. Overall, the sunlit near side exhibits a more pronounced polarization to solar illumination, which is closely related to its higher FeO abundance.

Although the average DOP of the far side is comparable to that of the near side, the surface composition of the far side is more heterogeneous, influenced by long-term geological evolution, meteorite impacts, and other factors. Moreover, due to the current lack of direct polarization measurements for the far side, despite the model having demonstrated high reliability on the near side, its extrapolation to the far side may amplify errors and increase uncertainty in DOP estimation. Consequently, the estimation of far-side DOP is affected by a combination of factors, including model applicability, instrument precision, and solar illumination. Future acquisition of polarization measurements specifically targeting the lunar far side would significantly enhance our understanding of polarization characteristics across the entire lunar surface.

## 5. Discussion

This study addresses a long-standing gap in lunar far-side polarization research by establishing a linear regression model between FeO abundance and the DOP, which estimates the DOP distribution of the lunar far side. This is particularly significant given that prior studies have predominantly focused on the near side [15,50,51,52,53], and systematic polarization observations of the far side, which are critical for capturing the Moon’s full geological diversity and evolutionary history, have remained scarce. The reason for this is the lack of relevant observational data. The Chang’e-4 mission accomplished the first soft landing on the lunar far side and provided the first in situ regolith temperature measurements [54]. The Chang’e-6 mission retrieved lunar samples from the far side for the first time [55,56]. In addition to these in situ investigations on the lunar far side, KPLO is currently the only mission equipped with a polarization imaging camera for lunar observations. These missions have not yet provided systematic polarization information. Our global DOP distribution thus serves as a foundational dataset for investigating geological and structural differences between the near and far sides, complementing existing spectral and compositional data to depict a more comprehensive picture of lunar surface heterogeneity and advancing a unified understanding of the Moon’s surface properties.

The observed hemispheric differences in DOP further underscore the scientific value of our results, as they likely reflect variations in regolith maturity and space weathering exposure. FeO abundance, a representative chemical component of lunar regolith, directly links to surface material sources, regolith maturity, and space weathering intensity. Existing studies have demonstrated that low-maturity regolith, which is characterized by coarser grains, lower nanophase iron abundance, and higher bedrock-like reflectance, corresponds to lower DOP values, thereby establishing polarization data as a unique tool for assessing regolith maturity [57,58]. This is a critical complement to spectral measurements, as it provides physical information that spectral data alone cannot fully capture, opening new avenues for quantifying space weathering effects across the lunar surface.

Nevertheless, this study has several limitations that must be considered when interpreting the results. First, while the FeO-DOP linear model demonstrates applicability and reliability, DOP is not exclusively determined by FeO abundance; it is also influenced by surface topography, image registration errors, and the quantity/quality of observational data. Second, the low spatial resolution of current polarization data prevents the assessment of pixel-level uncertainties, limiting the ability to resolve fine-scale geological features that may exhibit distinct DOP signatures. Third, the inherent differences in geological structure, surface morphology, and albedo between the near and far sides introduce biases when extrapolating near-side-derived models to the far side, potentially affecting the accuracy of far-side DOP predictions.

To gain further insights into the differences between the near and far sides of the Moon and their underlying causes, it is essential to develop observation platforms equipped with polarization imaging cameras to acquire direct measurements of far-side polarization. Missions such as KPLO could play a crucial role in validating far-side DOP predictions. We therefore recommend that future lunar exploration programs routinely incorporate polarimetric instruments.

## 6. Conclusions

In this study, we investigated the polarization characteristics of the lunar surface by establishing a quantitative relationship between the DOP and FeO abundance. By selecting control points directly from ground-based polarization images of the lunar near side, we developed a linear regression model that effectively characterizes the spatial distribution of observed DOP, particularly in regions with high FeO abundance and elevated DOP values.

Using this model, we derived the first global DOP distribution map of the Moon, including the previously unobservable far side. The results reveal notable hemispheric differences in polarization that are closely linked to variations in FeO abundance.

Additionally, we analyzed the variation in DOP with lunar phase angle and found that the fitted results are highly consistent with actual observations in both trend and magnitude. This confirms the reliability of the model and indicates the feasibility of applying ground-based polarimetric data to large-scale lunar remote sensing.

In summary, this study proposes a new method for indirectly obtaining polarization information of the lunar far side. It not only provides an effective approach to infer the polarization characteristics of the lunar far side but also offers new insights into the comprehensive evaluation of the overall polarization state of the Moon. Moreover, the inferred far-side DOP distribution provides a means to assess lunar regolith maturity, offering insights into the exposure age of regions and informing theories of lunar evolution.

## Figures and Tables

**Table 1:** Lunar landing site information, including longitude, latitude, and DOP values sourced from [11,39,48], alongside the corresponding fitted DOP derived in this study.

Site	Longitude	Latitude	DOP	Fitted DOP
Apollo 11-average (in the literature)	23.473° E	0.673° N	0.109	0.091
luna16-average (in the literature)	56.364° E	0.514° S	0.107	0.098
luna20-average (in the literature)	56.624° E	3.786° N	0.056	0.030
Apollo 15 average	3.638° E	26.132° N	0.074	0.061
Apollo 16 average	15.504° E	8.973° S	0.040	0.022
Apollo 17 average	30.766° E	20.192° N	0.066	0.062
CE-5	51.916° W	43.057° N	0.108	0.095
Apollo 11-average (in the literature)	23.473° E	0.673° N	0.109	0.091

## Data Availability

The data presented in this study are available upon request from the corresponding author.
